# Elevated ASCL1 activity creates de novo regulatory elements associated with neuronal differentiation

**DOI:** 10.1186/s12864-022-08495-8

**Published:** 2022-04-03

**Authors:** Laura M. Woods, Fahad R. Ali, Roshna Gomez, Igor Chernukhin, Daniel Marcos, Lydia M. Parkinson, Ahmad N. Abou Tayoun, Jason S. Carroll, Anna Philpott

**Affiliations:** 1 Department of Oncology, University of Cambridge, Cambridge, UK; 2 Wellcome-MRC Cambridge Stem Cell Institute, Jeffrey Cheah Biomedical Center, Cambridge Biomedical Campus, Cambridge, UK; 3 College of Medicine, Mohammed Bin Rashid University of Medicine and Health Sciences, Dubai, United Arab Emirates; 4 Center for Genomic Discovery, Mohammed Bin Rashid University of Medicine and Health Sciences, Dubai, United Arab Emirates; 5 Cancer Research UK Cambridge Institute, University of Cambridge, Cambridge, UK; 6 Al Jalila Genomics Center, Al Jalila Children’s Hospital, Dubai, United Arab Emirates

**Keywords:** ASCL1, Reprogramming, Neuroblastoma, Induced neurons, Differentiation

## Abstract

**Background:**

The pro-neural transcription factor ASCL1 is a master regulator of neurogenesis and a key factor necessary for the reprogramming of permissive cell types to neurons. Endogenously, ASCL1 expression is often associated with neuroblast stem-ness. Moreover, ASCL1-mediated reprogramming of fibroblasts to differentiated neurons is commonly achieved using artificially high levels of ASCL1 protein, where ASCL1 acts as an “on-target” pioneer factor. However, the genome-wide effects of enhancing ASCL1 activity in a permissive neurogenic environment has not been thoroughly investigated. Here, we overexpressed ASCL1 in the neuronally-permissive context of neuroblastoma (NB) cells where modest endogenous ASCL1 supports the neuroblast programme.

**Results:**

Increasing ASCL1 in neuroblastoma cells both enhances binding at existing ASCL1 sites and also leads to creation of numerous additional, lower affinity binding sites. These extensive genome-wide changes in ASCL1 binding result in significant reprogramming of the NB transcriptome, redirecting it from a proliferative neuroblastic state towards one favouring neuronal differentiation. Mechanistically, ASCL1-mediated cell cycle exit and differentiation can be increased further by preventing its multi-site phosphorylation, which is associated with additional changes in genome-wide binding and gene activation profiles.

**Conclusions:**

Our findings show that enhancing ASCL1 activity in a neurogenic environment both increases binding at endogenous ASCL1 sites and also results in additional binding to new low affinity sites that favours neuronal differentiation over the proliferating neuroblast programme supported by the endogenous protein. These findings have important implications for controlling processes of neurogenesis in cancer and cellular reprogramming.

**Supplementary Information:**

The online version contains supplementary material available at 10.1186/s12864-022-08495-8.

## Background

ASCL1 (Achaete-Scute complex homolog 1) is a master regulator of neurogenesis and plays an important role in the development of both the central and peripheral nervous systems [1]. In the central nervous system, ASCL1 regulates fate determination of both neuronal and glial lineages by inducing the differentiation of GABAergic inhibitory interneurons and oligodendrocytes, while inhibiting astrocyte differentiation [2–5]. In the peripheral nervous system, the transient expression of ASCL1 in neuronal precursor cells and its subsequent downregulation is required for the differentiation of sympathetic neuronal progenitor cells [6–9]. In contrast, expression of ASCL1 can induce cell cycle exit and differentiation of neuronal stem cells [10]. ASCL1 also has an essential role in regulating differentiation within other lineages. For example, ASCL1 has been shown to regulate pulmonary neuroendocrine differentiation, where genetic ablation of ASCL1 has a negative effect on the differentiation of lung neuroendocrine cells [11, 12].

In addition to being a key transcriptional regulator during embryogenesis and in adult stem cells, a role for ASCL1 has been demonstrated in a range of solid neuronal and neuroendocrine tumours such as glioblastoma, neuroblastoma, small cell lung cancer and prostate cancer [13–19]. In neuroblastoma, ASCL1 is a member of the core transcriptional regulatory circuit that maintains the adrenergic (ADRN) phenotype, an aggressive subtype of neuroblastoma. ASCL1 expression is modulated by the transcription factors LMO1 and MYCN and together they regulate expression of other ADRN-associated factors such as TBX2, GATA3 and PHOX2B [19].

ASCL1 belongs to the basic helix-loop-helix (bHLH) family of transcriptional regulators controlling multiple targets involved in cell proliferation, differentiation, and maturation [20, 21]. Interestingly, ASCL1 can promote both proliferation and differentiation of cells within the same lineage [21], although the mechanisms for the switch in cell fate are not well understood. Indeed, this dual activity of ASCL1 may underpin regulatory mechanisms that control the switching from proliferation to differentiation of neural progenitors.

As befits its role as a master regulator of neurogenesis, a number of studies have looked at genome-wide binding and gene activation by ASCL1 in several contexts [18, 21, 22]. Cognate binding sites of ASCL1 are predominantly found in distal enhancer regions of target genes and binding mostly results in activation of its gene targets. However, ASCL1-mediated gene repression has also been reported [23]. An interesting characteristic of ASCL1 is its ability to open up repressed chromatin regions and activate transcription of neuronal genes. This chromatin opening has been proposed to be associated with ASCL1 binding at sites with a specific combination of histone marks, identifying ASCL1 as an “on-target” pioneer factor that can reprogram cells from multiple lineages, as well as pluripotent adult and embryonic stem cells, to neuronal cells usually in combination with co-factors [1, 24–26]. However, reprogramming is not always efficient, varying considerably between individual cells. In addition, cells can be erroneously redirected to other lineages, ultimately affecting the overall efficiency of neuronal reprogramming. Recent single-cell analysis studies determined that the intercellular heterogeneity associated with fibroblast reprogramming by ectopic ASCL1 can be mainly attributed to the redirection of cells towards an alternate myogenic fate [27]. Sustained expression of ASCL1 is most likely required for efficient reprogramming as well as neuronal differentiation.

In previous studies, we have shown that post-translational modifications of ASCL1 such as phosphorylation modulates its level and activity, ultimately controlling ASCL1’s ability to drive reprogramming of ectoderm to neurons as well as differentiation of neural progenitors and neuroblastic cancer cells [8, 13, 28]. In particular, neuroblastoma cells respond to un(der)phosphorylated ASCL1 by down-regulation of a neuroblastic core regulatory circuit of transcription factors accompanied by cell cycle exit and re-engagement of a genome-wide programme of neuronal differentiation [13]. To better understand how ASCL1 expression level and phosphorylation status controls chromatin target binding genome-wide, we have used the neuroblastoma cell line SH-SY5Y (termed NB cells below) as a model representing a broadly permissive neurogenic environment.

We find that increasing ASCL1 levels in NB cells results in engagement of a large number of new low-affinity binding sites and extensive changes in gene expression associated with both neuronal and myogenic pathways. Moreover, we use a phospho-mutant form of ASCL1 to show that preventing ASCL1 post-translational modification further enhances its binding at numerous chromatin sites, and this is accompanied by increased gene activation and repression that is associated with neuronal differentiation. Understanding genome-wide responses to changes in ASCL1 levels and phosphorylation status has significant implications for its use in approaches to direct cell fate and manipulation of its function in cancer cells and reprogramming protocols.

## Results

### ASCL1 overexpression in a neurogenic environment leads to promiscuous target binding and gene regulation

In fibroblasts, ASCL1 acts as a key "on-target" pioneer factor to open up closed chromatin and drive reprogramming to neurons [25]. In neuronally committed NB cells, endogenous ASCL1 binds promoter, enhancer and super-enhancer regions, supporting proliferation and maintaining the mutually regulated core transcriptional circuit that is a signature of adrenergic-type neuroblastic tumours [19]. We have previously shown that increasing the activity of ASCL1 by ectopic overexpression of a hyper-active form of ASCL1 that cannot be phosphorylated on 5 serine-proline sites (S-A ASCL1) [28] drives differentiation of NB cells [13]. Using NB cells as a broadly permissive neurogenic environment, we set out to investigate the effects of increasing ASCL1 activity by overexpression and inhibition of phosphorylation on its genome-wide binding and subsequent gene activation. We used a doxycycline-inducible lentiviral system to induce the overexpression of wildtype (WT) or S-A ASCL1 in NB cells, which we have described previously [13]. Using this system, *ASCL1* mRNA expression levels increased 2–12 fold compared to endogenous *ASCL1* [13], and this was translated in the amount of ASCL1 protein present (Supplementary Fig. 1A). In comparison, the level of ASCL1 overexpression achieved by inhibition of the NOTCH signalling pathway in glioblastoma cells is between 1.5–2.5 fold [17].

**Fig. 1:**
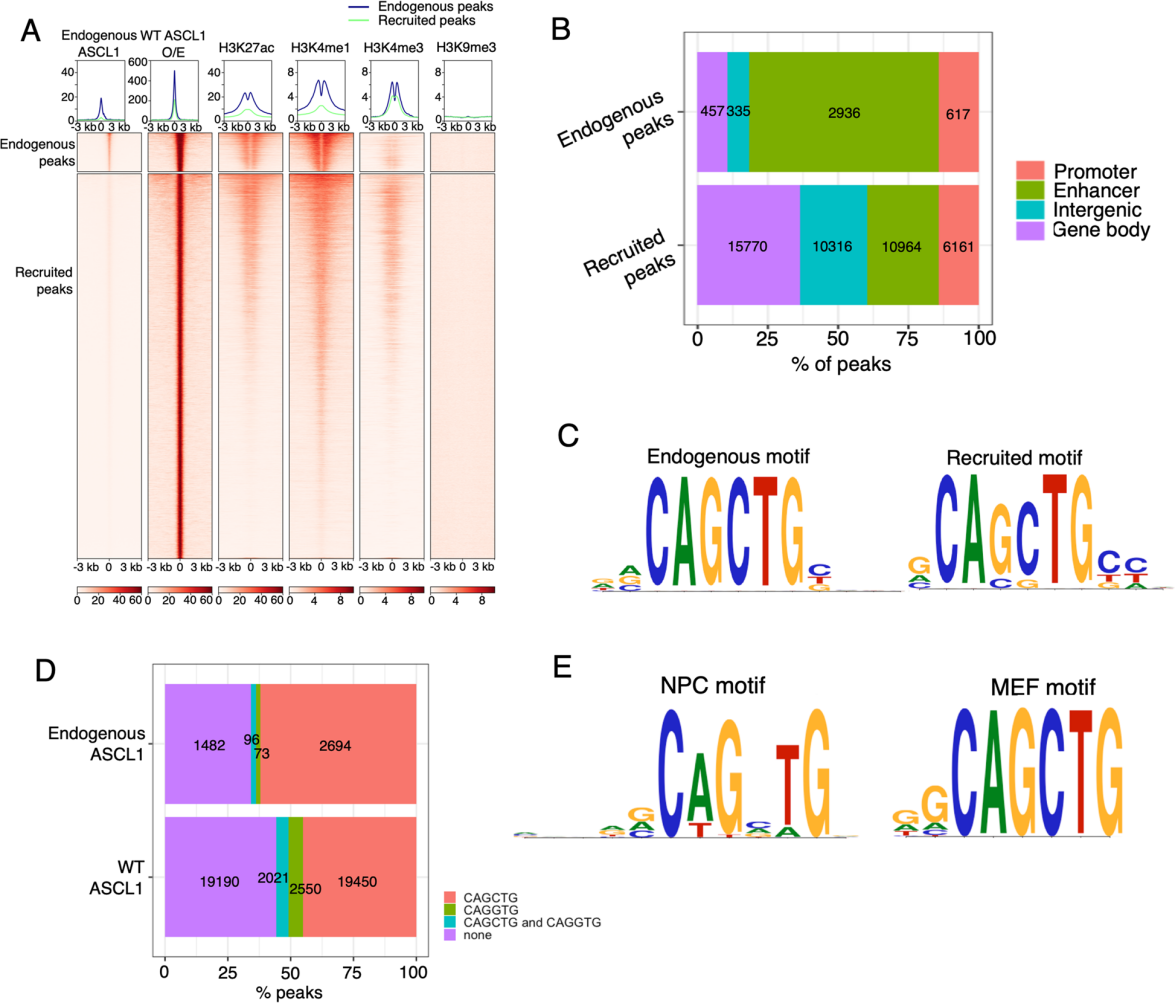
ASCL1 overexpression leads to association with de novo binding sites. **a** Average profile (top) and aligned heatmap (bottom) showing relative levels of endogenous and overexpressed WT ASCL1 binding at ASCL1 peaks identified in NB cells with endogenous ASCL1, or with overexpressed ASCL1 after 24 h induction with 1 μg/ml doxycycline (*n* = 8). Recruited peaks were called only in overexpressed conditions, whereas endogenous peaks were present in both endogenous and overexpressed conditions. Shown alongside, endogenous histone modification data from SH-SY5Y cells (H3K27ac, H3K4me1, H3K4me3, H3K27me3) or LAN6 cells (H3K9me3) [30, 31]. Data shown ± 3 kb from the peak centre. **b** Barchart showing the percentage of endogenous and recruited peaks that fall within NB gene promoters, enhancers, intergenic regions, and gene bodies. Actual number of peaks shown in each bar. **c** Top E-box binding motif identified in ASCL1 peaks with endogenous (endogenous motif), or overexpressed ASCL1 (recruited motif), against a background of random sequences matched for GC content. **d** Stacked barplot showing the proportion of endogenous and recruited ASCL1 peaks containing the E-box motifs CAGCTG and/or CAGGTG, or neither motif. Actual number of peaks shown in each segment. **e **Top E-box binding motif identified in top 1000 ASCL1 peaks from mouse NPC neuronal precursor cells (NPCs) and mouse embryonic fibroblasts (MEFs) induced for ASCL1 overexpression [25], against a background of random sequences matched for GC content

First, we wished to determine whether increasing ASCL1 protein over endogenous levels in a neurogenic environment leads to enhanced binding at sites occupied by endogenous ASCL1, new binding at previously unoccupied sites, or both. To do this, we performed ChIP-seq for ASCL1 protein under endogenous conditions and after overexpression of WT ASCL1. ASCL1 ChIP-seq was carried out in a total of 3 biological replicates for cells expressing endogenous levels of ASCL1 (i.e. ASCL1-inducible but not treated with doxycycline), and 4 replicates each from two separate clones after 24 h of WT ASCL1 induction. Reads from the replicates were merged and peaks called on the single merged libraries, prior to merging there was a high correlation of ASCL1 ChIP-seq signal between biological replicates (Supplementary Fig. 1B). We first compared endogenous ASCL1 binding to that of ectopically overexpressed WT ASCL1 (Fig. 1A) in NB cells engineered to overexpress ASCL1 in response to doxycycline [13]. After ASCL1 overexpression, ectopic ASCL1 binds strongly to endogenous sites and is also recruited to many previously un-bound sites (recruited sites). The majority (68%) of endogenous sites are found in enhancer regions, as defined by publicly available H3K27ac profiles of NB cells [29], while approximately 15% are in promoter regions (Fig. 1B). ASCL1 response genes with promoters within 3 kb of an endogenous or recruited ASCL1 binding site showed a similar pattern of transcriptional regulation after ASCL1 induction, indicating that recruited ASCL1 binding sites act as functional regulatory elements (Supplementary Fig. 1C).

We performed motif discovery analysis on sites bound by endogenous ASCL1, which confirmed a strong preference for the canonical E-box motif 5'-CAGCTG-3' (Fig. 1C) that has been associated with ASCL1 activity in development of the mouse ventral telencephalon [21], as well as binding during ASCL1-mediated reprogramming in fibroblasts and differentiating neural stem cells [1, 25]. At recruited sites, we hypothesised that over-expressed ASCL1 might bind to more degenerate E-box motifs. Indeed, recruited sites show increased degeneracy at the central two nucleotides, with the consensus sequence 5'-CASSTG-3' (S: G/C nucleotides) (Fig. 1C).

We performed further analysis using HOMER differential motif discovery to find motifs differentially enriched between endogenous and recruited sites. This showed that recruited sites are enriched for a non-canonical E-box motif 5’-CAGGTG-3’, whereas the canonical 5’-CAGCTG-3’ motif is the most enriched motif at endogenous peaks (Supplementary Fig. 1D). Approximately 60% (2694/4345) of endogenous ASCL1 binding was centred within 200 bp of a canonical motif, compared to only 45% (19,450/43211) of recruited peaks. In contrast, recruited peaks were more likely to contain the non-canonical motif, or neither motif, and to have a greater number of motifs per peak, pointing to potentially lower-affinity binding of recruited sites when high levels of ASCL1 are present (Fig. 1D, Supplementary Fig. 1E). We hypothesized that this increased degeneracy of ASCL1 binding at high levels of ASCL1 in this neurogenic environment could either be determined by a generally permissive cellular context, or it may reflect saturation of endogenous high fidelity binding sites and utilization of lower affinity sites in the presence of excess ASCL1. We investigated these possibilities using publicly available ASCL1 ChIP-seq data from mouse neuronal precursor cells (NPCs) and mouse embryonic fibroblasts (MEFs) rendered inducible for ASCL1 overexpression [25]. We performed motif discovery analysis of the top 1000 ASCL1 binding sites in neuronal (NPCs) and non-neuronal (MEFs) contexts to identify the consensus sequence for ASCL1 binding in these different cell types. Interestingly, overexpressed ASCL1-bound to a strict canonical E-box motif in MEFs, whereas in NPCs ASCL1 was able to bind more promiscuously (Fig. 1E), suggesting it is the neuronal context that allows degenerate ASCL1 binding and utilization of non-canonical E-box motifs, rather than simply an excess of ASCL1 protein.

### Dephosphorylation of ASCL1 further enhances genome-wide binding

We have previously shown that preventing phosphorylation of ASCL1 by mutating all potential cyclin-dependent kinase target serine-proline sites to alanine-proline (S-A ASCL1) enhances its ability to drive neuronal differentiation in developing *Xenopus* embryos [28]. S-A ASCL1 expression also enhances morphological differentiation of reprogrammed fibroblasts when compared to expression of WT ASCL1 [28]. Moreover, S-A ASCL1 drives differentiation of NB cells by promoting downregulation of key proliferative targets and upregulation of CDK inhibitors and differentiation genes [13]. Considering the enhanced pro-differentiation activity of S-A ASCL1 in these different contexts, we next set out to interrogate differences in genome-wide binding of phospho-mutant S-A and WT ASCL1 in NB cells.

**Fig. 2:**
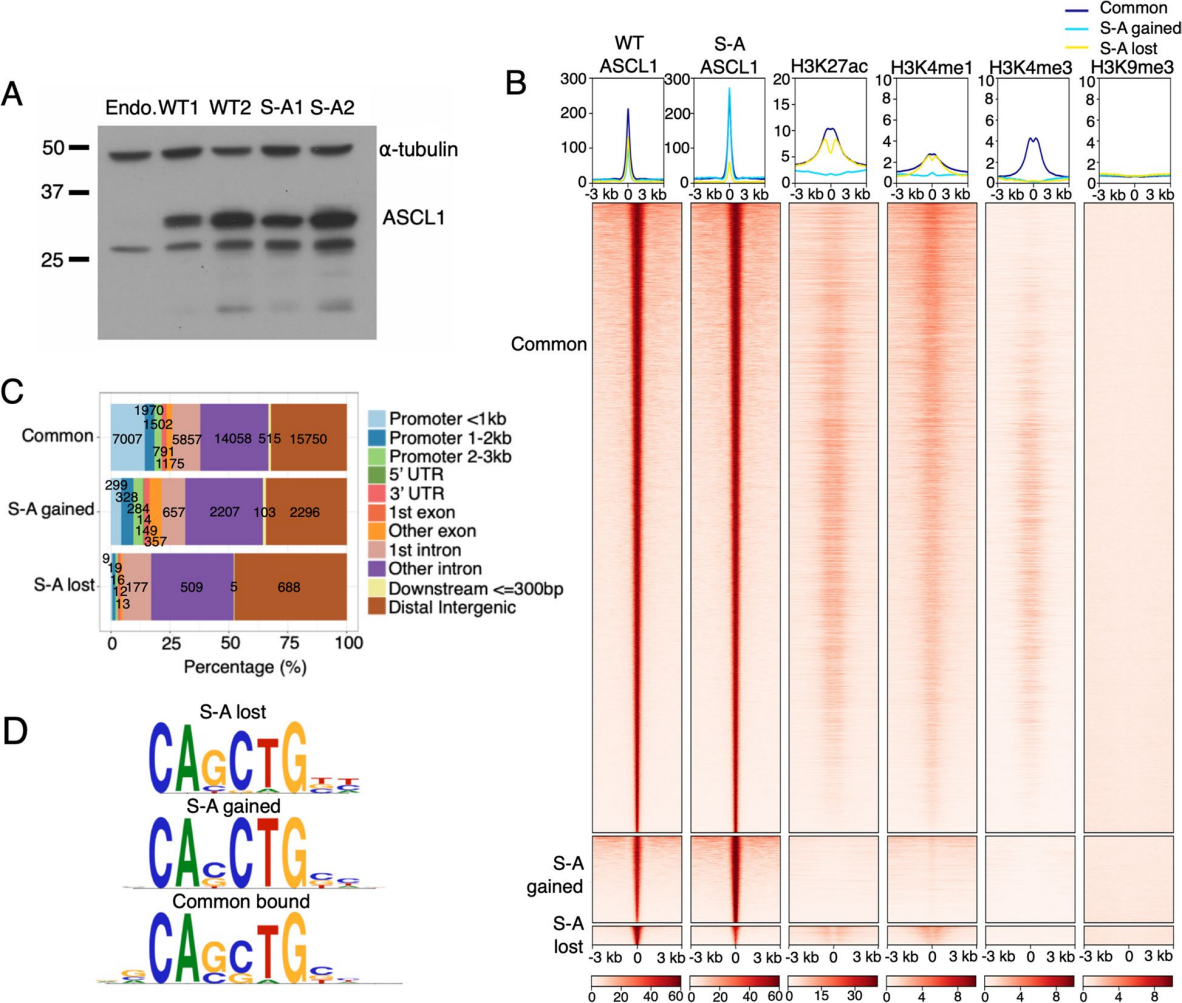
Dephosphorylation of ASCL1 further enhances its genome-wide binding. **a** Western blot showing overexpression of WT and S-A ASCL1 in two pairs of unique clones after 24 h induction with 1 μg/ml doxycycline, α-tubulin loading control. Endo. = endogenous. Full-length blot is presented in Supplementary Fig. 6. **b** Average profile (top) and aligned heatmap (bottom) showing WT and S-A ASCL1 binding (*n* = 8) at common, S-A gained, and S-A lost peaks, alongside endogenous histone modification data from SH-SY5Y cells (H3K27ac, H3K4me1, H3K4me3, H3K27me3) or LAN6 cells (H3K9me3) [30, 31]. Data shown ± 3 kb from the peak centre. **c** Stacked barplot showing the distribution of ASCL1 common, S-A gained, and S-A lost peaks in genomic features. Actual numbers of peaks shown in each segment. **d** Top enriched E-box motif identified in common, S-A gained, and S-A lost ASCL1 peaks, against a background of random sequences matched for GC content

In order to distinguish differences in genome-wide binding that are specifically due to differences in ASCL1 phosphorylation rather than ASCL1 protein level, we generated two pairs of WT and S-A expressing NB clones. Each pair was matched for ASCL1 protein level, with one pair, WT1 and S-A1 expressing a low level of ectopic WT or S-A ASCL1, while the other pair, WT2 and S-A2 expressed a matched but higher level (Fig. 2A, Supplementary Fig. 1A). Combined data from WT1 and WT2 were compared with combined data from S-A1 and S-A2, which allows us to identify specific differences that can be attributed to changes in phosphorylation of ASCL1 rather than to differences in protein level. All subsequent analysis was performed on this combined dataset. To confirm phosphorylation status of ASCL1 in our inducible cell lines, we performed western blotting for WT and S-A ASCL1 with and without phosphatase treatment, separated on SDS-PAGE gels containing Phos-tag™ reagent. WT ASCL1 migrates more slowly than S-A ASCL1 only in the absence of phosphatase, confirming that WT ASCL1 is readily phosphorylated in NB cells (Supplementary Fig. 2A).

To understand how ASCL1 phosphorylation may be influencing its genome-wide chromatin binding, we used our ASCL1 ChIP-seq data to generate a list of ASCL1 binding peaks and performed differential binding analysis to identify regions where ASCL1 binding is significantly affected by its phosphorylation status. While WT and S-A ASCL1 both bound at a similar level at the large majority of sites, their chromatin association differed at some regions (Fig. 2B). The majority of these phospho-regulated sites were specific to the ASCL1 overexpression state, and only 149/8164 overlapped with endogenous ASCL1 binding sites (Supplementary Fig. 2B). These sites were disproportionately found in introns and distal intergenic regions suggestive of enhancer binding, but were rarely found in promoters (Fig. 2C). We split these differentially bound regions into two categories: peaks where S-A ASCL1 binding was significantly higher than WT (S-A gained) and peaks where S-A ASCL1 binding was significantly lower than WT (S-A lost) (Fig. 2B). S-A gained and S-A lost regions showed a subtly different binding motif preference compared to regions that bind WT and S-A ASCL1 equally (Fig. 2D).

Using publicly available datasets of epigenetic marks in SH-SY5Y NB cells [30, 31], common WT and S-A ASCL1 binding sites overwhelmingly showed the enhancer marks H3K27ac and H3K4me1, with a subset also being marked by H3K4me3 (Fig. 2B), consistent with its known enhancer binding activity [1, 32, 33]. However, S-A gained sites were not marked with these common activating histone modifications, suggesting that un(der)phosphorylated ASCL1 can bind to inactive/closed chromatin not accessed by endogenous ASCL1 (Fig. 2B). ASCL1 was previously described to bind a trivalent chromatin signature in MEFs (H3K4me1, H3K27ac, and H3K9me3), which is associated with permissiveness for ASCL1-mediated “on-target” pioneer activity and induction of neuronal fate [25]. We investigated whether phospho-regulated ASCL1 binding sites were marked with this trivalent signature. Interestingly we saw no evidence of the trivalent signature in any ASCL1-targeted regions (Fig. 2B).

### Genome-wide transcriptional changes in a neurogenic environment mediated by ectopic ASCL1

In NB cells, endogenous ASCL1 acts to maintain stemness by promoting cell growth while suppressing neuronal differentiation [19]. However, other studies have shown that high level ASCL1 overexpression can force neuronal differentiation in neural stem cells [10], and overcome epigenetic barriers to reprogram terminally differentiated cells to neurons [34]. Furthermore, upregulating ASCL1 levels by NOTCH inhibition has been shown to differentiate glioblastoma stem cells to a neuronal fate [17]. We have previously shown that ASCL1 overexpression leads to down-regulation of specific cell-cycle activators and core regulatory circuit transcription factors that maintain the adrenergic phenotype of NB cells, as well as up-regulation of some CDK inhibitors and pro-differentiation factors in this context [13]. Looking beyond the specific circuitry and regulators known to control the phenotypic behaviour of NB cells, we wanted to determine the broader global changes brought about by ASCL1 overexpression in this permissive cell type.

**Fig. 3:**
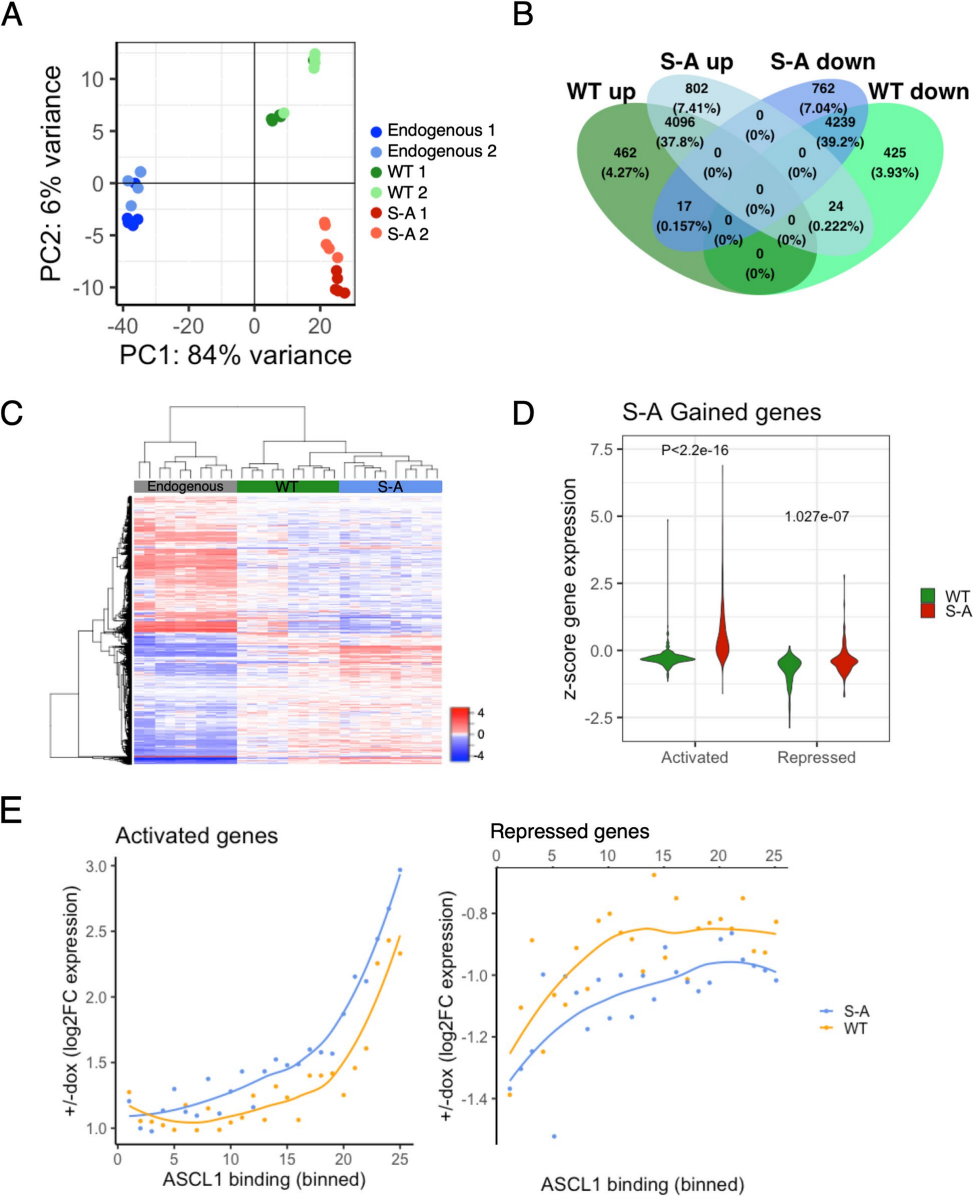
Genome-wide transcriptional changes after ectopic ASCL1 expression in NB cells. **a** PCA of RNA-seq data from NB cells expressing uninduced levels of ASCL1 (endogenous), or overexpressing WT or S-A ASCL1 after 24 h induction with 1 μg/ml doxycycline (*n* = 10). Data from two separate clones is shown for each condition. **b** Venn diagram showing the number of WT or S-A ASCL1 response genes which are up- or down-regulated compared to WT ASCL1 uninduced cells after 24 h induction with 1 μg/ml doxycycline (DESeq2 padj < 0.05, *n* = 10), and the number of target genes which are shared by WT and S-A ASCL1. Proportion and actual numbers of genes shown in each segment. **c** Heatmap showing relative expression of genes significantly differentially expressed between WT uninduced with endogenous levels of ASCL1, and WT and S-A ASCL1 induced NB clones after 24 h induction with 1 μg/ml doxycycline (DESeq2 padj < 0.05, *n* = 10). Z-scaled RPKM values for endogenous WT uninduced (grey), WT induced (green), and S-A induced (blue) clones are shown. **d** Violin plot showing relative expression of WT or S-A ASCL1 target genes associated with significantly increased binding of S-A ASCL1 compared to WT (DESeq2 padj < 0.05, *n* = 8). Genes are grouped on the x-axis by gene activation or repression. Significance between WT and S-A groups was determined by two-tailed unpaired t-test. **e** Dotplot showing the correlation between average logFC gene expression (y axis), and average ASCL1 binding strength (x axis) in NB cells overexpressing WT or S-A ASCL1 after 24 h induction with 1 μg/ml doxycycline. Points represents equal sized groups of ASCL1 response genes with an ASCL1 binding site within 3 kb of the promoter, divided into bins based on ASCL1 binding strength. Activated genes (left), repressed genes (right)

To compare the transcriptome-wide effects of ectopic WT and S-A ASCL1 on gene expression, we performed principal component analysis (PCA) on RNA-seq data of NB cells expressing uninduced levels of ASCL1, in comparison to cells ectopically expressing doxycycline-induced WT or S-A ASCL1 for 24 h. We found that overexpression of both WT and phospho-mutant ASCL1 had a pronounced effect on the transcriptome (Fig. 3A). In addition to transcriptional changes resulting from increased overall ASCL1 levels, PCA also clearly demonstrated a marked difference in transcriptional response between WT and S-A ASCL1-overexpressing cells. This indicates that both ASCL1 level and its post-translational modification has a significant effect on transcriptional activation genome-wide. As doxycycline itself has been shown to induce gene expression changes in mouse renal epithelial cells, pterygium, and rat aortic tissue, [35–37] and can activate a dopaminergic differentiation programme [38], we sequenced additional RNA libraries generated from parental NB cells either untreated or treated with 1 μg/ml doxycycline for 24 h. Compared to doxycycline-induced overexpression of ASCL1, doxycycline treatment of parental cells caused minimal changes in gene expression (Supplementary Fig. 3A), indicating that our findings can be attributed to ASCL1 induction rather than doxycycline itself.

To investigate the effects on downstream target regulation that can be attributed to differences in ASCL1 phosphorylation, we performed differential expression analysis using DESeq2 to compare uninduced cells with cells overexpressing either WT or S-A ASCL1. We identified 8376 genes which were differentially regulated by both WT and S-A ASCL1 when compared to expression in parental cells, while 887 genes were differentially regulated only by WT, and 1564 only by S-A ASCL1 compared to parental cells (Fig. 3B, C). Activated genes were associated with synaptic assembly and signalling, whereas repressed genes were enriched for terms relating to DNA replication (Supplementary Fig. 3B).

Combining our ChIP-seq and RNA-seq analyses, we investigated whether increased WT or S-A ASCL1 binding has different consequences for associated gene activation. To associate an ASCL1 peak to its putative target we considered the ASCL1 peaks identified in Fig. 2B and assigned to each peak the closest ASCL1 response gene, within a distance cut-off of 3 kb. When there was similar binding of WT and S-A ASCL1, there was a small but statistically significant amplification of gene regulation by the dephosphorylated protein (Supplementary Fig. 3C). Where there was a significant increase in S-A ASCL1 binding compared to WT, (S-A gained), the difference was more pronounced and nearby genes tended to be up-regulated to a greater extent by phospho-mutant ASCL1 than WT ASCL1 (Fig. 3D). Importantly, this points to a direct correlation between extent of ASCL1 chromatin association that is influenced by ASCL1 phosphorylation binding and gene expression, rather than indicating phospho-regulated recruitment of other transcription factors by ASCL1 that indirectly drive the enhanced gene activation seen with S-A ASCL1 compared to WT ASCL1. We then looked at P300 binding over differentiation targets previously shown to be more strongly activated in response to dephosphorylated ASCL1 [13], and found that binding of the histone acetyltransferase P300 was significantly increased after induction of the S-A compared to WT, suggesting enhanced histone acetylation and activation of these genes (Supplementary Fig. 3D).

ASCL1 has previously been described as a transcriptional activator in neural progenitor cells, with little evidence for direct repression in this context [1]. As we found that approximately 50% of ASCL1 responsive genes were repressed in NB cells after 24 h of ASCL1 induction (Fig. 3B), we wanted to investigate the mechanism of ASCL1-mediated gene repression in this system. Increased strength of ASCL1 binding was positively correlated with target gene activation indicating direct regulation (Fig. 3E, left panel). However, we saw no correlation between ASCL1 binding strength and repression, suggesting ASCL1-mediated gene repression is indirect (Fig. 3E, right panel). Interestingly, even when repressed target promoters were found within 1 kb of ASCL1-bound regions, the strongest repression was generally associated with the lowest levels of ASCL1 binding, consistent with the possibility that low ASCL1 is permissive for binding of transcriptional repressors [39, 40].

### WT ASCL1 supports neuroblast proliferation while un(der)phosphorylated ASCL1 promotes neurogenesis

**Fig. 4:**
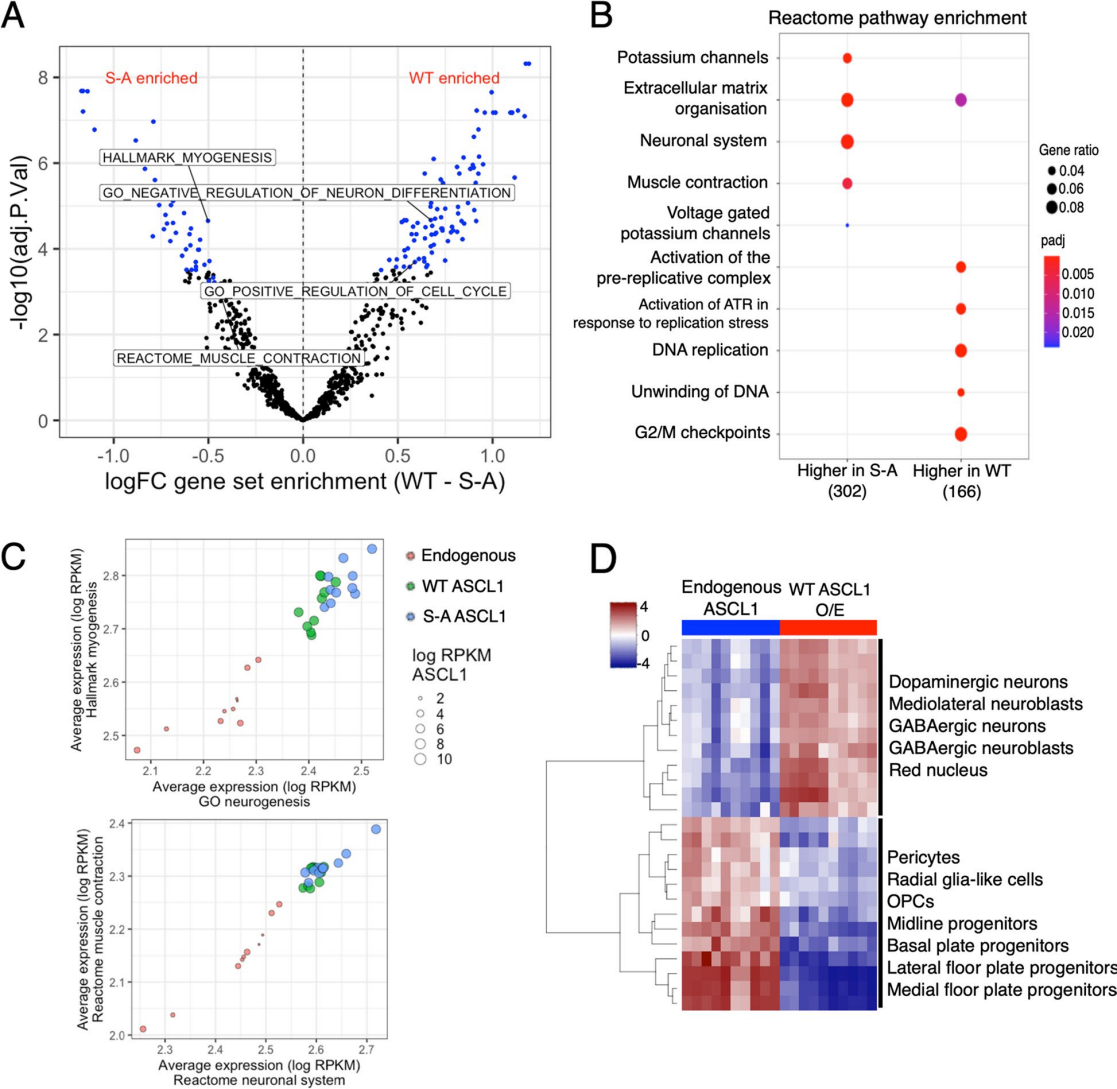
WT ASCL1 supports cell proliferation while un(der)phosphorylated ASCL1 promotes neurogenesis. **a** Volcano plot showing gene set variation analysis (GSVA), comparing RNA-seq data from WT and S-A ASCL1 overexpressing cells. Significantly differentially enriched terms highlighted in blue, terms of particular interest are labelled (padj < 0.05, *n* = 10) Gene sets preferentially enriched after WT or S-A ASCL1 induction have positive or negative enrichment values, respectively. **b** Dotplot showing significantly overrepresented Reactome Pathway gene sets. Gene sets were tested against genes significantly differentially expressed between WT and S-A ASCL1 overexpressing NB cells (DESeq2 padj < 0.05, *n* = 10). Total number of genes in each group shown in brackets. Point colour = Benjamini–Hochberg adjusted p-value, point size = gene ratio (number of genes related to term/total number of significant genes). **c** Dotplots showing the correlation of averaged expression of genes in the GO neurogenesis, and Hallmark myogenesis gene sets (left), or Reactome muscle contraction and neuronal system gene sets (right). Red = endogenous WT uninduced ASCL1, green = WT ASCL1, blue = S-A ASCL1. Point size indicates log2 RPKM ASCL1 expression, each point represents one biological replicate (*n* = 10). **d** Heatmap showing neuronal cell type marker gene sets (Broad Institute C8 set) found to be differentially enriched between WT uninduced and WT ASCL1 induced expression datasets (*n* = 10), by gene set variation analysis (GSVA)

We next sought to identify pathways that were differentially regulated by ectopic expression of WT and S-A ASCL1, comparing gene set variation analysis (GSVA) and pathway overrepresentation analysis for WT and S-A ASCL1-overexpressing NB cells. WT ASCL1-induced genes were enriched for cell cycle and DNA replication terms, whereas S-A ASCL1-induced genes were associated with the neuronal system (Fig. 4A, B), in line with our previous findings showing that phospho-mutant ASCL1 binding is reduced at cell cycle regulators and increased at differentiation markers, and accompanied by gene repression or activation, respectively [13]. For example, dephosphorylated ASCL1 binds less strongly than the phosphorylated form to the promoter region of cell cycle regulator and serine/threonine kinase Polo Like Kinase 2 (PLK2) [41], abolishing ASCL1 associated PLK2 activation (Supplementary Figs. 4A,B). Conversely, S-A ASCL1 binds more strongly than WT to multiple regions within the putative neuronal differentiation effector WNT9A [42], and correlates with greater WNT9A upregulation (Supplementary Fig. 4A, C). This is in line with our previous findings, that ASCL1 dephosphorylation suppresses proliferation and promotes neuronal differentiation in both developing embryos and NB cells [8, 13, 28].

Interestingly, S-A ASCL1 targets are also preferentially enriched for genes related to myogenesis and muscle contraction (Fig. 4A), reminiscent of muscle gene activation seen by ASCL1 overexpression in fibroblasts [43]. S-A ASCL1 binds more strongly than WT within muscle regulator KLF15 [44], which is activated by S-A but not WT ASCL1 induction (Supplementary Figs. 4A, D). The ASCL1 consensus DNA binding sequence is closely related to that of MYOD1, a master regulator of the muscle lineage. Hence ASCL1 and MYOD1 overlap significantly in their target binding, despite driving different transcriptional outputs and cell identities [45]. We therefore wanted to directly investigate whether S-A ASCL1 was erroneously activating muscle genes in NB cells, leading to lower fidelity neuronal cell identity compared to WT ASCL1. As a measure of neuronal and muscle cell identity, we defined neuronal and muscle gene signatures based on publicly available gene annotation databases and calculated the average expression of these gene signatures in each sample. Both WT and S-A ASCL1 induction led to up-regulation of muscle and neuronal gene signatures (Fig. 4C). S-A ASCL1 induced greater activation of both gene signatures. However, there was no clear bias towards either cell identity as a result of different ASCL1 phosphorylation status, indicating that de-phosphorylation of ASCL1 enhances the magnitude of response to ectopic expression but does not affect the specificity of the transcriptional response.

In vivo, ASCL1 is expressed in the central nervous system in ventral progenitors of the telencephalon leading to the GABAergic fate, as well as during the differentiation of multiple other neuron subtypes including those of the peripheral nervous system (PNS) [46]. NB cells are derived from the PNS and are usually committed to the noradrenergic sympathetic lineage. This led us to investigate how the promiscuous binding and gene activation we observed with overexpression of ASCL1 in NB cells affects the expression of neuronal subtype markers in this otherwise lineage-restricted cell type. Using gene set variation analysis (GSVA) we looked for differential enrichment of neuronal cell type signature gene sets between uninduced and doxycycline-induced overexpression of ASCL1. This showed that uninduced levels of ASCL1 promote expression of neuronal precursor gene sets, whereas ASCL1 overexpression activates genes associated with GABAergic and dopaminergic fates (Fig. 4D).

## Discussion

ASCL1 is a pro-neural transcription factor and a master regulator of neurogenesis that is critical to the development of both central and peripheral nervous systems [22]. During normal embryonic development ASCL1 is expressed transiently, and its transcriptional activity is modulated across the different stages of development. ASCL1 has also been implicated as an oncogene or tumour suppressor in multiple cancers [14–16, 18, 19]. For instance, ASCL1 has been shown to be critical in maintaining “stemness” in human glioblastoma, and when upregulated by NOTCH inhibition, ASCL1 promoted glioblastoma cell differentiation into a neuronal lineage attenuating its tumorigenicity [17]. In addition, we have recently shown that upregulating ASCL1 levels in NB cells suppresses the expression of core transcriptional regulators that are required for NB proliferation while simultaneously driving NB cells towards differentiation [13].

In addition to its role in neurogenesis and cancer, ASCL1 has emerged as a key component in transcription factor cocktails that can be used to reprogramme a variety of cells into neurons. However, the efficiency of reprogramming varies considerably between different cell types as well as between individual cells of the same type [47, 48] and is often accompanied by the activation of aberrant programmes, which are likely to interfere with efficient production of reprogrammed neurons [27]. In this context, reprogramming efficiency seems to be largely determined by the presence of repressors or co-factors and the epigenetic profile of the recipient cells. Single cell RNA-seq analysis of the reprogramming event identified induction of an aberrant myogenic program along with silencing of the transgene ASCL1 as factors that affect the reprogramming efficiency of ASCL1 in fibroblasts [27, 43].

In a reprogramming context, ASCL1 binding causes genome-wide chromatin remodelling at around 12 h after induction followed by nucleosome phasing, subsequent to the enhancer activation observed during reprogramming [43]. A trivalent epigenetic chromatin signature (H3K4me1, H3K27ac, and H3K9me3) was identified at ASCL1 binding sites in cell types that are permissive for reprogramming, such as mouse embryonic and human dermal fibroblasts, but absent in restrictive cells such as human keratinocytes. ASCL1 is sufficient to initiate reprogramming, while other transcriptional regulators, in this case BRN2 and MYT1L, are required in conjunction with ASCL1 to engage the full neuronal programme and bring about terminal differentiation [25]. Methods to enhance both the efficiency and the uniformity of ASCL1-mediated reprogramming are of great importance and this requires a better understanding of the parameters that govern ASCL1s association with target genes in both permissive and non-permissive environments.

In this study, we first investigated the genome-wide effects of increasing expression of ASCL1 in a NB cell line that is already specified as neurogenic, and permissive for neuronal differentiation. After WT ASCL1 induction, we saw increased binding at sites also occupied by endogenous ASCL1 as well as new binding at a large number of recruited sites. Changes in binding were associated with changes in the expression of thousands of genes as befits ASCL1’s role as a transcriptional master regulator of neurogenesis. Consistent with previous studies, we find that ASCL1-mediated gene upregulation is generally direct, whereas we see little evidence for direct ASCL1 gene-mediated repression which is instead likely mediated by downstream effectors of ASCL1 (Fig. 3E) [1].

When the transcriptional consequences of ASCL1-mediated reprogramming of fibroblasts have previously been analysed at a single cell level, strong upregulation of a myogenic programme was often observed, generally resulting in abortive reprogramming [27]. Even in the neurogenic environment of NB cells, we saw significant activation of genes associated with myogenesis. In common with findings in fibroblasts, promiscuous binding of ASCL1 after ectopic expression is likely to occur on recruited sites that are also targets of the potent myogenic bHLH factor MYOD1 that binds a similar E-box motif to ASCL1, resulting in upregulation of muscle genes and pathways [27, 43, 45]. This demonstrates that the epigenetic and co-factor landscape of cells that are already committed to a neural lineage is not enough to constrain the response to increased levels of ASCL1, at least at 24 h after induction as used here. Indeed, in this neurogenic environment, overexpression of ASCL1 led to recruitment of new and lower affinity DNA binding sites compared to endogenous levels of the protein. Interestingly, the non-canonical CAGGTG E-box motif that we found to be enriched in the newly recruited sites associated with ASCL1 overexpression has previously been reported in nucleosome-occupied closed sites in embryonic stem cells, while more open nucleosome-depleted sites contained the canonical E-box motif, that we find is preferentially bound by endogenous ASCL1 [49]. These recruited sites were also less likely to be marked by activating histone modifications (Fig. 1A), consistent with ASCL1’s ability to act as pioneer factor binding to and opening up closed chromatin when expressed at a high level. Also, it is striking that the neurogenic environment afforded by NB cells and NPCs appears to be more permissive for overexpressed ASCL1 binding to non-canonical motifs than fibroblasts (Fig. 1C, E). This is consistent with a NB cell chromatin state being more open to ASCL1-dependent modulation, because here ASCL1 can bind at low affinity sites compared to less responsive cell types such as fibroblasts, which are likely to require a greater enhancement of ASCL1 activity to elicit a reprogramming response. During reprogramming of fibroblast cells with a cocktail of factors including ASCL1, a myogenic fate can be initially induced only to disappear at later time points upon induction of MYT1L, a repressor of alternative fate pathways [45, 50]. In our data, MYT1L is upregulated on ASCL1 overexpression and this may act to prevent muscle identity in NB cells (Supplementary Fig. 4E).

We have previously shown that ASCL1 phosphorylation plays an important role in modulating ASCL1 activity [13, 28]. Cyclin dependent kinases and RAS/ERK mediated signalling can phosphorylate ASCL1 on multiple serine/proline sites [8, 28, 51]. Phosphorylated forms of ASCL1 favour progenitor maintenance, while un(der)phosphorylated ASCL1 inhibits proliferation and enhances differentiation [13, 28]. Notably, genes that are bound to same extent by WT and phospho-mutant ASCL1, are regulated similarly by both proteins, while differences in transcriptional activity depends on phospho-dependent gain or loss of binding (Fig. 3D, Supplementary Fig. 3C. This clearly indicates that phosphorylation works by controlling ASCL1 binding to DNA rather than by phospho-dependent recruitment of additional co-factors. Phosphorylation of ASCL1 does not greatly affect promoter binding and activation but instead changes binding to intronic and intergenic regions of the genome, which are free of activating histone modifications under endogenous conditions, suggesting they have a closed chromatin conformation (Fig. 2B), and ASCL1 phosphorylation is able to control its pioneer activity.

In contrast to gene activation, gene repression was correlated with low ASCL1 binding within 1 kb of the target gene promoter, suggesting ASCL1 mediated repression is generally an indirect effect. At the small number of genes where S-A ASCL1 overexpression leads to loss of ASCL1 binding and gene repression, (so called S-A lost genes), target downregulation may not be by direct repression but instead represent a loss of ASCL1-mediated activation (Fig. 3E, Supplementary Fig. 3C). It is also possible that there is active gene repression by factors that can only bind when ASCL1 is displaced, as previously observed in primary myoblasts and rhabdomyosarcoma where SNAI1/2 occupy and block MYOD access to differentiation targets, in order to control entry into myogenic differentiation [39, 40]. Indeed, H3K27ac and H3K4me1 profiles show a biphasic distribution surrounding the ASCL1 binding site at S-A lost regions, suggesting that ASCL1 is bound there under endogenous conditions and may be displaced by transcriptional repressors upon ASCL1 overexpression (Fig. 2B).

## Conclusions

Taken together, our results support a model where the absolute activity of ASCL1 in a given context determines whether it supports progenitor maintenance, neuronal differentiation and/or aberrantly activates alternative pathways such as myogenesis. In this model, at low levels of activity, ASCL1 favours binding at high affinity canonical sites found in genes associated with proliferating neuroblasts. As activity increases additional lower affinity and sometimes non-canonical sites are recruited, which are associated with a programme of neuronal differentiation, while feedback mechanisms may suppress genes that would otherwise promote progenitor maintenance [13]. Increasing activity may also result in binding at sites that are associated with activating alternative developmental pathways. However, this may trigger further feedback and feed-forward mechanisms, for instance involving MYTL1-mediated repression of alternative fates [50], to maintain neurogenic specificity. Here we describe two ways of enhancing ASCL1 activity: a simple increase in protein level, and preventing multi-site phosphorylation. ASCL1 expression is also known to oscillate in neural stem cells but becomes stable and higher at the onset of neuronal differentiation. This transition occurs in co-ordination with the downregulation of HES1, which would otherwise mediate repression of ASCL1 activity via the NOTCH pathway [52, 53]. Multiple mechanisms of controlling ASCL1 activity is consistent with a central role of its overall activity in determining whether ASCL1 supports a proliferating progenitor programme or drives differentiation. Defining how ASCL1 levels and post-translational modification alters the strength and fidelity of genome-wide activity is of upmost importance if we are to optimize protocols for generation of pure populations of reprogrammed neurons in vitro and in vivo.

NB cells express endogenous ASCL1, which has an important function in maintaining the proliferating neuroblastic phenotype [19]. However, this endogenous level of expression must be tightly regulated as increasing ASCL1 leads to dramatic changes genome-wide and functionally results in a pronounced switch in tumour cell behaviour towards a less proliferative and more differentiated state [13]. The relative plasticity of the neurogenic chromatin of NB cells in response to changes in ASCL1 activity opens up the possibility of enhancing endogenous ASCL1 function by dephosphorylation, to promote differentiation for therapeutic benefit in NB. ASCL1 acts as an oncogene and/or tumour suppressor in a variety of other cancers including small cell lung cancer, glioblastoma, and advanced prostate cancer. In all these tumours, manipulating ASCL1 activity could alter the balance between a proliferating progenitor tumour cell identity, usually associated with a poorer prognosis, and that of a more differentiated cell type where prognosis may be better [12–14, 28, 54–57].

## Methods

### Cell Culture

As described previously [13]. Briefly, the NB cell line SH-SY5Y (kindly gifted by Prof. John Hardy, UCL) used in experiments was verified by submitting genomic DNA for short tandem repeat (STR) sequencing and compared to the Children’s Oncology Group Cell Line Identification database (http://www.cogcell.org/clid.php) to ensure it was genetically matched to standardised cell lines. SH-SY5Y cells were cultured in DMEM/F-12 with GlutaMAX™ supplement, 10% tetracycline-free Fetal Bovine Serum (Clontech, cat# 631,106), and 100 units/ml penicillin and 100 μg/ml streptomycin.

### Generation of Lentivirally Transduced Cell Lines

To generate stably transduced NB cell lines, SHSY-5Y cells were infected with viral particles encoding either human WT or S-A ASCL1 alongside Tet-On transactivator at a multiplicity of infection (MOI) of 10. Selected cells were clonally expanded and tested for ASCL1 overexpression. For detailed method, please refer to associated file at https://www-ncbi-nlm-nih-gov.ezproxy.u-pec.fr/geo/query/acc.cgi?acc=GSE153823.

### Western blotting and Phos-tag™ western blot

ASCL1 expression was induced with 1 μg/ml doxycycline for 24 h and cell were lysed with RIPA buffer (50 mM Tris, pH 8.0, 150 mM NaCl, 1.0% IGEPAL® CA-630 (Sigma), 0.5% sodium deoxycholate, 0.1% SDS and protease inhibitor cocktail). Protein concentration was determined using BCA protein assay kit (Thermo, cat# 23,225). For western blotting, SDS-PAGE electrophoresis was carried out using 4–20% Criterion™ TGX™ Precast Midi Protein Gel (Biorad #5,671,094). Total protein extract was loaded and transferred to nitrocellulose membrane and blocked with 5% milk 0.1% TBS-Tween 20 for 1 h, before probing with anti-ASCL1 1 in 1000 (Abcam, ab74065). or anti-α-tubulin 1 in 5000 (Abcam ab7291). For Phos-tag™ western blot, phosphatase treatment was performed by 30 min incubation at 30 °C with 400 units of Lambda Protein Phosphatase (NEB, cat# P0753S). Phosphatase treated samples were run on 8% acrylamide gels polymerised with 20 μM Phos-tag™ reagent (WAKO, cat# AAL-107) and 40 μM MnCl2. After running and before transfer, Phos-tag™ gels were washed three times, 10 min each with transfer buffer (25 mM Tris–HCl, 190 mM glycine, 20% methanol) plus 10 mM EDTA, followed by a final wash with transfer buffer.

### RNA-seq

For detailed experimental methods please see https://www-ncbi-nlm-nih-gov.ezproxy.u-pec.fr/geo/query/acc.cgi?acc=GSE153823. Briefly, RNA-sequencing experiments were performed in SH-SY5Y, WT and S-A ASCL1 tetracycline-inducible stable cell lines, with two clones of each type. For endogenous ASCL1 levels (referred to as endogenous), WT ASCL1 tetracycline-inducible cells were cultured without doxycycline and RNA extracted. For ASCL1 overexpressing samples, cells were induced with 1 µg/ml doxycycline for 24 h and RNA extracted. Experiment was performed in at least five biological replicates for each cell line, resulting in 10 replicates for each condition. Single-end 50 bp reads were generated on the Illumina HiSeq 2000 sequencer and aligned to the human genome version hg19 using STAR 2.5.1a [58].

### RNA-seq analysis

Differential expression analysis was performed using DESeq2 version 1.14.1 [59]. WT and S-A ASCL1 lines were analysed in parallel, comparing untreated (endogenous) and doxycycline-induced samples (*p*-adjusted value < 0.05, *n* = 10) to identify genes regulated by WT or S-A ASCL1. In Fig. 3B, WT and S-A datasets were directly compared to identify differentially expressed genes (padj < 0.05, *n* = 10). Heatmaps were generated using Heatmap.2 in R, using z-scaled RPKM values. Pathway overrepresentation analysis was performed with ClusterProfiler [60], using the ReactomePA package in R [61]. Gene set variation analysis (GSVA) was done using the R GSVA package [62], testing Broad Institute C8 cell type, Hallmark, GO, and Reactome Pathway gene set databases [63]. PCA analysis of RNA-seq data was performed in R, on rlog transformed counts using the top 500 most variable genes.

### ChIP and ChIP-seq

ChIP and ChIP-seq were performed as described previously [13] using anti-ASCL1 (Abcam, cat# ab74065) anti-P300 (Santa-Cruz, cat# sc-585), and anti-IgG (Abcam cat# ab6706). Detailed experimental methods are available at https://www-ncbi-nlm-nih-gov.ezproxy.u-pec.fr/geo/query/acc.cgi?acc=GSE153823. ChIP-seq experiments were performed in two separate clones each of SH-SY5Y WT or S-A ASCL1 tetracycline-inducible cells, induced with 1 μg/ml doxycycline for 24 h, or for endogenous levels of ASCL1, one clone of WT ASCL1 inducible cells cultured with no doxycycline treatment (referred to as endogenous). Experiments were performed in 4 biological replicates per clone for WT and S-A ASCL1 overexpressing samples (*n* = 8), and 3 biological replicates for endogenous ASCL1 (*n* = 3). ChIP-seq and input libraries were prepared using the ThruPLEX® DNA-seq Kit (Rubicon Genomics). Reads were mapped to the hg19 genome using Bowtie2 version 2.2.6, aligned reads with the mapping quality less than five were filtered out. The read alignments from three (endogenous ASCL1) or eight (overexpressing ASCL1) replicates were combined into a single library, and peaks were called with model-based analysis for ChIP-seq 2 (MACS2) version 2.0.10.20131216 using sequences from SH-SY5Y chromatin extracts as a background input control. WT and S-A ASCL1 peaks were merged into one consensus peak list, and split into 3 groups: common (FDR > 0.05), S-A gained (FDR < 0.05, log2FC > 1), and S-A lost with DiffBind version 2.14.0 [64] using the DESeq2 method (FDR < 0.05, log2FC > 1).

### ChIP-seq analysis

Tag density and average trend plots were generated on RPKM normalised bigwig files, using Deeptools computeMatrix, plotHeatmap version 3.4.1 [65]. Motif analysis was performed using the HOMER findMotifsGenome.pl command [66], using either default GC-normalised background sequences, or a specified list of GC-normalised background sequences when finding differentially enriched motifs between conditions. Analysis of ASCL1 binding correlation with gene up/downregulation was performed by splitting ASCL1 response genes with an ASCL1 binding site within 1 kb of their TSS into 25 equal bins, based on the level of ASCL1 binding in that region, and plotting against the average log2 fold change of genes within that bin, between uninduced and dox-induced cells.

## Supplementary Information

**Additional file 1.**

## Data Availability

The ASCL1 ChIP-seq and RNA-seq datasets generated and/or analysed during the current study are accessible through GEO series accession number GSE153823 (https://www-ncbi-nlm-nih-gov.ezproxy.u-pec.fr/geo/query/acc.cgi?acc=GSE153823). Publicly available histone modification ChIP-seq data are available under GEO accession GSE80197 (https://www.ncbi.nlm.nih.gov/geo/query/acc.cgi?acc=GSE80197) (H3K4me3, H3K27ac, H3K4me1, H3K27me3; all from SH-SY5Y cells) [30], and GSE70920 (https://www.ncbi.nlm.nih.gov/geo/query/acc.cgi?acc=GSE70920) (H3K9me3 from LAN6 cells) [31]. ASCL1 ChIP-seq data from MEFs and NPCs can be found under accession GSE43916 (https://www.ncbi.nlm.nih.gov/geo/query/acc.cgi?acc=GSE43916) [25].

## Abbreviations

ASCL1: Achaete-Scute complex homolog 1
NB: Neuroblastoma
bHLH: Basic helix loop helix
WT: Wildtype
S-A: Serine to alanine
PCA: Principal component analysis
NPC: Neuronal precursor cell
MEF: Mouse embryonic fibroblast
GSVA: Gene set variation analysis
PNS: Peripheral nervous system
