# Supplementary material for: Elevated ASCL1 activity creates de novo regulatory elements associated with neuronal differentiation
Source: BMC Genomics. 2022 Apr 3;23:255. doi: 10.1186/s12864-022-08495-8 (PMC8977041; doi:10.1186/s12864-022-08495-8)
Supplement: Supplementary file 1 — Additional file 1. [file 12864_2022_8495_MOESM1_ESM.pdf]

Supplementary figure 1

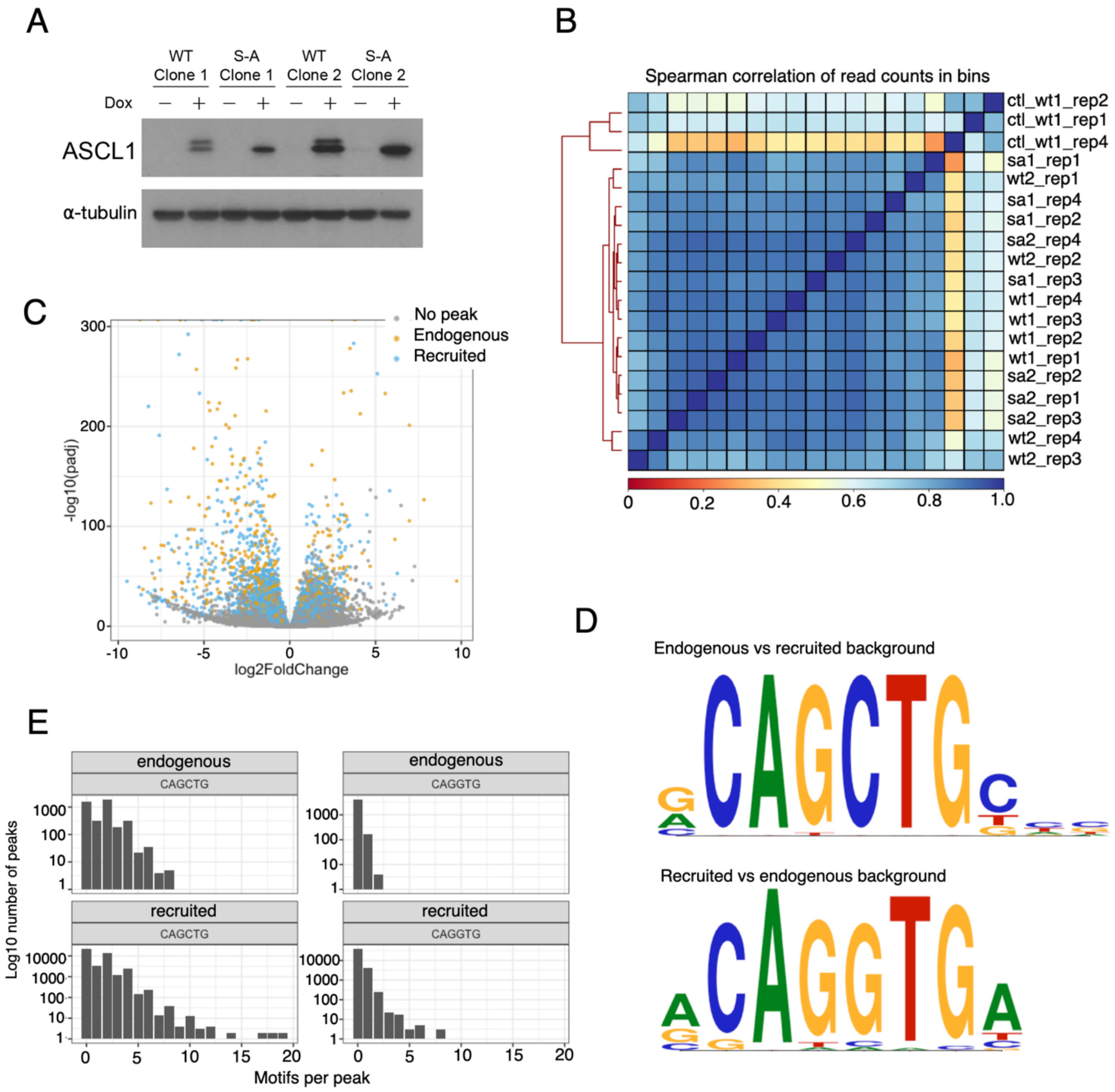

a) Western blot showing ASCL1 protein levels in lentivirally transduced NB cells, with and without induction of WT or S-A ASCL1 by 24 hours of treatment with 1 μg/ml doxycycline. α-tubulin loading control. Full length blot shown in Supplementary Figure 5.

b) Spearman's correlation of ASCL1 ChIP-seq signal in 10 kb running bins along the genome.

c) Volcano plot showing changes in gene expression after 24 hours of WT ASCL1 induction with 1 μg/ml doxycycline, compared to endogenous WT uninduced. Genes

with endogenous (orange) or recruited (blue) ASCL1 binding within 3 kb of their promoter are highlighted. Genes with ASCL1 no peak within 3 kb in grey.

d) Top enriched E-box binding motif identified NB cell ASCL1 peaks with endogenous or overexpressed WT ASCL1, when compared against a background of overexpressed WT ASCL1 or endogenous ASCL1 peaks, respectively, matched for GC content.

e) Histograms showing the distribution of endogenous and recruited ASCL1 peaks containing multiple CAGCTG or CAGGTG E-box motifs.

Supplementary figure 2

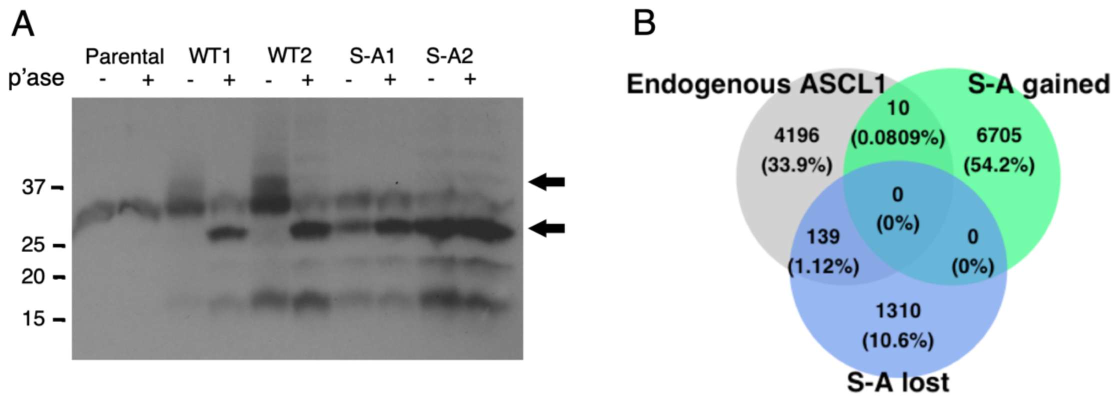

a) Phos-tag™ SDS-PAGE of SH-SY5Y parental, WT ASCL1 overexpressing, or S-A ASCL1 overexpressing cells, protein extracts, with or without lambda phosphatase (p'ase) treatment to remove protein phosphorylation. Probed for ASCL1 protein, top arrow indicates highly phosphorylated ASCL1, bottom arrow indicates un(der)phosphorylated ASCL1. Full-length blot is presented in Supplementary Figure 7.

b) Venn diagram showing the overlap between endogenous ASCL1 binding sites, and those differentially bound between WT and S-A ASCL1 overexpressing cells (S-A gained and S-A lost).

Supplementary Figure 3

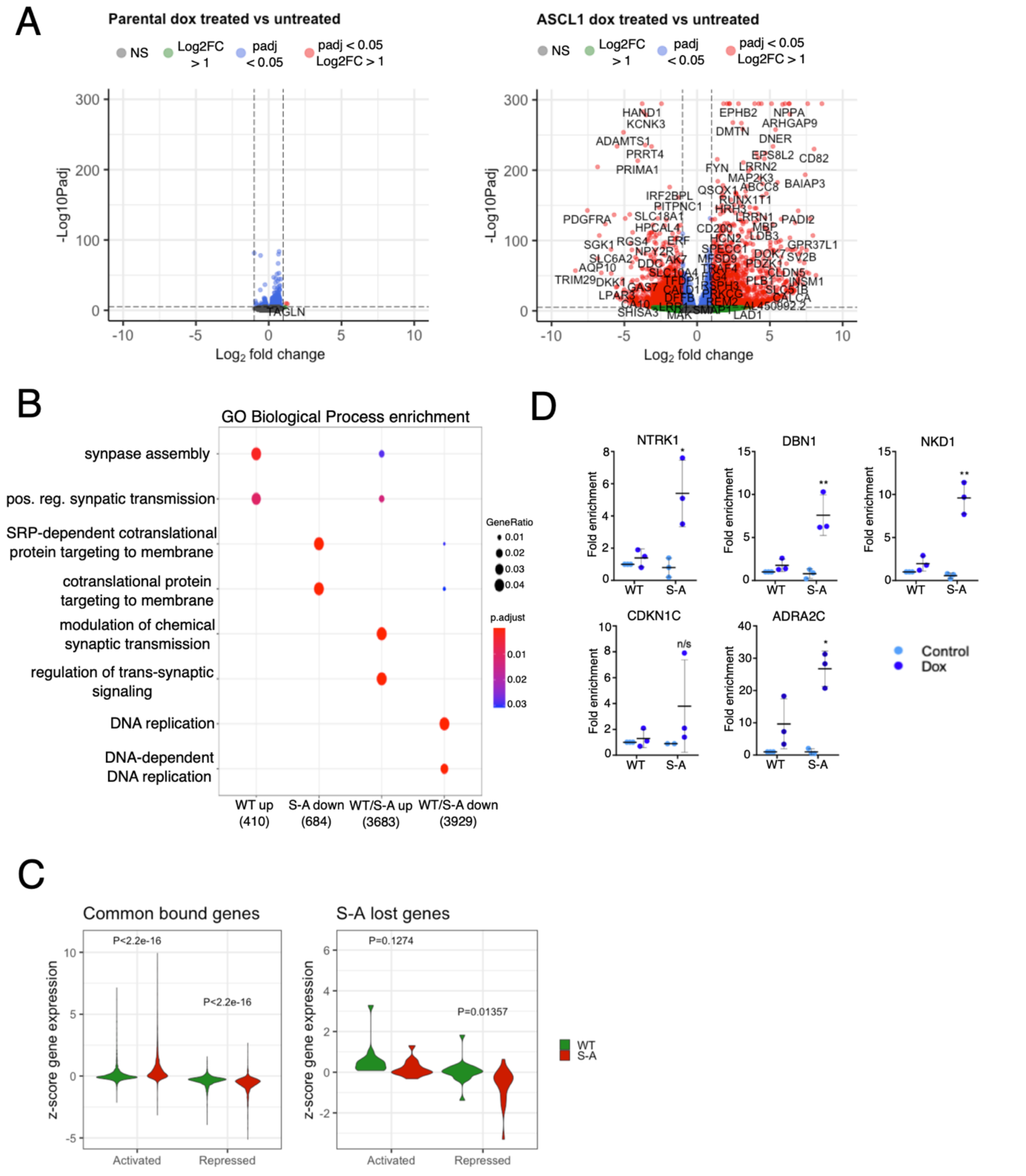

a) Volcano plots of RNA-seq data from parental NB cells either untreated or treated with 1 μg/ml doxycycline for 24 hours (left panel), and WT ASCL1 clones either untreated or induced with 1 μg/ml doxycycline for 24 hours (right panel). Highlighted

genes reaching DESeq2 p adjusted value < 0.05 (blue), log2 fold change > 1 (green), both (red).

b) Dotplot showing significantly overrepresented gene ontology biological process terms. Gene sets were tested against the groups represented in Figure 3B. Only groups in which significantly enriched terms were identified are shown. Total number of genes in each group shown in brackets. Point colour = Benjamini-Hochberg adjusted p-value, point size = gene ratio (number of genes related to term/total number of significant genes).

c) Violin plots showing relative expression of WT or S-A ASCL1 target genes associated with no significant difference in binding of S-A ASCL1 compared to WT (DESeq2 padj > 0.05, n = 8, left panel), or with significantly decreased binding of S-A ASCL1 compared to WT (right panel). Genes are grouped on the x-axis by gene activation or repression. Significance between WT and S-A groups was determined by two-tailed unpaired t-test.

d) ChIP-qPCR of P300 at selected differentiation targets in NB cells, either uninduced (Control) or 24 hours post-induction of WT and S-A ASCL1. n = 3, Mean ± SEM Significance between WT and S-A groups was determined by two-tailed unpaired t-test (*p < 0.05, ** p < 0.01).

Supplementary Figure 4

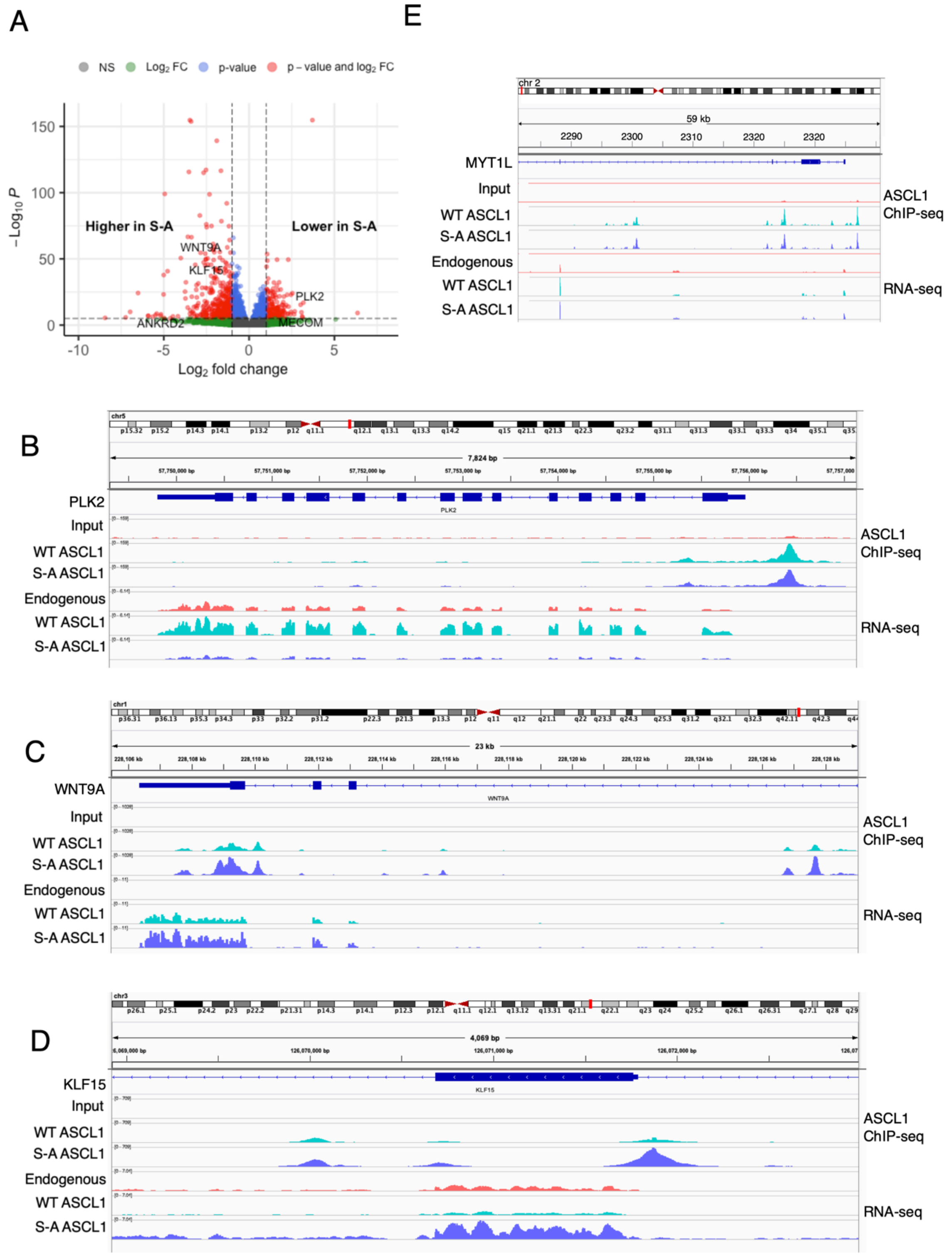

a) Volcano plot of comparing WT and S-A ASCL1 overexpression, with significantly differentially expressed genes highlighted (red, padj < 0.05, log2 fold change > 2.5). Selected genes annotated with GO terms related to cell cycle, neuronal

differentiation, and muscle differentiation are indicated. Highlighted genes reaching DESeq2 p adjusted value < 0.05 (blue), log2 fold change > 1 (green), both (red).

b) IGV tracks showing RNA-seq and ASCL1 ChIP-seq signal at the PLK2 gene, in cells expressing endogenous WT uninduced levels of ASCL1, overexpressing WT ASCL1, or overexpressing S-A ASCL1.

c) IGV tracks showing RNA-seq and ASCL1 ChIP-seq signal at the WNT9A gene, in cells expressing endogenous WT uninduced levels of ASCL1, overexpressing WT ASCL1, or overexpressing S-A ASCL1.

d) IGV tracks showing RNA-seq and ASCL1 ChIP-seq signal at the KLF15 gene, in cells expressing endogenous WT uninduced levels of ASCL1 (red), overexpressing WT ASCL1 (green), or overexpressing S-A ASCL1 (blue). Numbers on the y axis indicate RPKM value.

e) IGV tracks showing RNA-seq and ASCL1 ChIP-seq signal at the MYT1L gene, in cells expressing endogenous WT uninduced levels of ASCL1, overexpressing WT ASCL1, or overexpressing S-A ASCL1.

Supplementary Figure 5

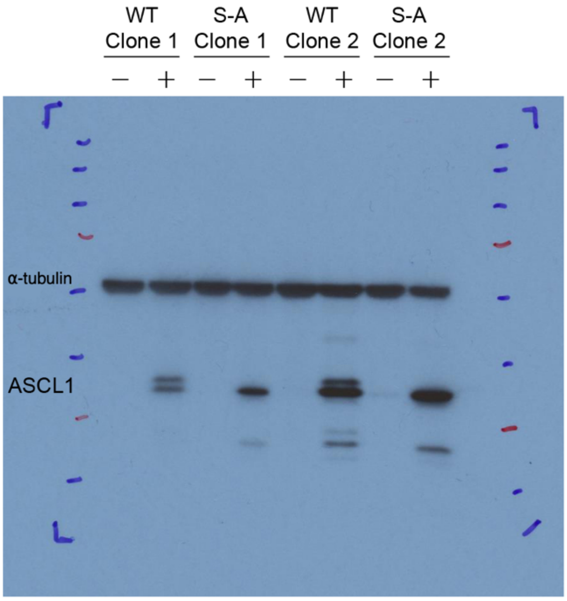

Full size western blot image shown in Supplementary Figure 1A. Western blot showing ASCL1 protein levels in lentivirally transduced NB cells, with and without induction of WT or S-A ASCL1 by 24 hours of treatment with 1 μg/ml doxycycline. α-tubulin loading control.

Supplementary Figure 6

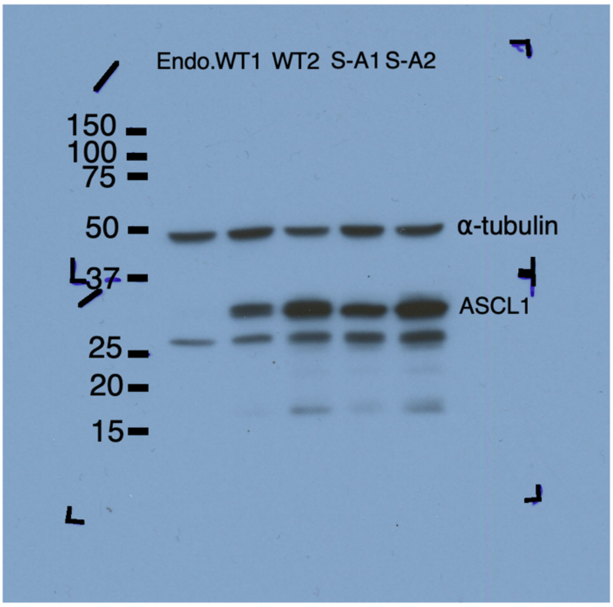

Full size western blot image shown in Figure 2A. Western blot showing endogenous ASCL1 in NB cells, and overexpression of WT and S-A ASCL1 in two unique clones each, after 24h induction with 1 μg/ml doxycycline. Diagonal and L-shaped markings indicate membrane boundaries. α-tubulin loading control.

Supplementary Figure 7

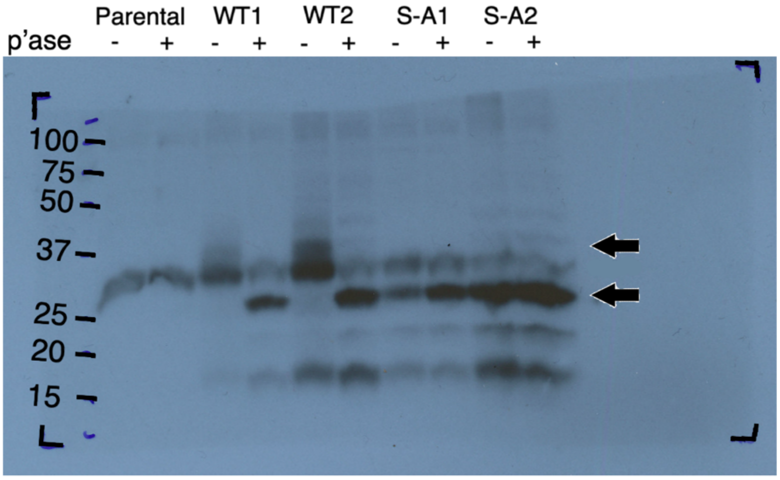

Full size western blot image shown in Supplementary Figure 2A. Phos-tag™ SDS-PAGE of protein extracts from NB parental, WT ASCL1 overexpressing, or S-A ASCL1 overexpressing cells induced with 1 μg/ml doxycycline for 24 hours, with or without lambda phosphatase (p'ase) treatment to remove ASCL1 phosphorylation. Probed for ASCL1 protein, top arrow indicates highly phosphorylated ASCL1, bottom arrow indicates un(der)phosphorylated ASCL1. L-shaped markings indicate membrane boundaries.
